# Preventing Musculoskeletal Strain in Dental Training: Effects of a Movement-Based Intervention

**DOI:** 10.3390/ijerph23060729

**Published:** 2026-05-30

**Authors:** Dorsaf Sahli, Carlos Oteo-Calatayud, Juan López-Quiles, Cristina Madrigal, Carmen López-Carriches, Pablo Revuelta-Cortés, Carlos Oteo-Morilla

**Affiliations:** Faculty of Dentistry, Universidad Complutense de Madrid, 28040 Madrid, Spain; dosahli@ucm.es (D.S.); oteoc@ucm.es (C.O.-C.); cmadrigal@odon.ucm.es (C.M.); maclopez@ucm.es (C.L.-C.); pabrev01@ucm.es (P.R.-C.); coteo@ucm.es (C.O.-M.)

**Keywords:** work-related musculoskeletal disorders, postgraduate dental students, occupational health, yoga, prevention, ergonomics, musculoskeletal health

## Abstract

**Highlights:**

**Public health relevance—How does this work relate to a public health issue?**

- Work-related musculoskeletal disorders are common among dental professionals and often begin during clinical training.
- This early exposure highlights a gap in preventive approaches within dental education.

**Public health significance—Why is this work of significance to public health?**

- This study examines a movement-based intervention implemented directly within postgraduate dental training.
- Addressing musculoskeletal health at this stage may help reduce cumulative occupational strain over time.

**Public health implications—What are the key implications or messages for practitioners, policy makers and/or researchers in public health?**

- Integrating movement-based interventions into dental training programmes offers a practical approach to support musculoskeletal health.
- Preventive strategies introduced during training may contribute to more sustainable clinical practice.

**Abstract:**

Work-related musculoskeletal disorders (WMSDs) are highly prevalent among dental professionals and often begin during clinical training. Preventive strategies within dental education remain limited. This study aimed to evaluate the effects of integrating a structured yoga-based programme into postgraduate dental training on musculoskeletal symptoms, perceived stress, and resilience. A randomized controlled pilot study was conducted in which participants were allocated to either an intervention group undertaking a 12-week yoga programme (one session per week) or a control group continuing usual clinical activities. Musculoskeletal symptoms were assessed using the adapted Nordic Musculoskeletal Questionnaire (NMQ) recall measure, while psychological outcomes were assessed using the Perceived Stress Scale (PSS-10) and the Connor–Davidson Resilience Scale (CD-RISC-10). Over the study period, musculoskeletal symptom scores decreased in the intervention group, whereas they remained relatively stable in the control group. Significant Group × Time interactions were observed for the primary musculoskeletal outcomes. No significant differences were found for work-related impairment, perceived stress, or resilience. These preliminary findings suggest that structured movement-based interventions may represent a potentially feasible preventive approach within dental training environments to address early occupational musculoskeletal strain. Further research with larger samples and longer intervention periods is needed to confirm these findings.

## 1. Introduction

Work-related musculoskeletal disorders (WMSDs) are highly prevalent among dental professionals due to prolonged static postures, repetitive movements, and the biomechanical demands of clinical practice. The dental profession is physically and mentally demanding, requiring sustained concentration, precision, and the ability to perform a wide range of therapeutic procedures under considerable time pressure and psychological strain [1]. Dentists work within the confined and often ergonomically challenging space of the oral cavity, where prolonged and delicate tasks generate significant physical workload, as clinical procedures are frequently carried out in asymmetric positions for extended periods [2]. Static muscular forces are considered more detrimental to the musculoskeletal system than dynamic forces [3] and, when combined with awkward postures such as neck flexion and rotation, forward inclination with loss of cervical and lumbar lordosis, trunk leaning toward the patient, and elevation of the arms, they promote muscular imbalance and further exacerbate mechanical stress on the musculoskeletal system [4].

In this regard, dental practice relies heavily on precise, small, and repetitive manual movements, often performed with fine and/or vibrating instruments [3]. Alexopoulos et al. [5] highlighted that this multifaceted physical demand constitutes a highly relevant occupational determinant in the development of musculoskeletal disorders (MSDs).

According to the World Health Organization (WHO), MSDs are defined as disorders of the muscles, tendons, peripheral nerves, or vascular system that do not result directly from an acute or instantaneous event [6]. Painful conditions and symptoms affecting the musculoskeletal system that originate from work-related movements are classified as work-related musculoskeletal disorders (WMSDs). Among dental professionals, these disorders represent one of the most prevalent occupational health problems and may appear early during professional training.

These problems are frequently associated with the continuous repetition of movements, often performed with considerable force and without sufficient recovery time [2]. Reported prevalence rates of musculoskeletal disorders in the literature vary widely, ranging from 63% to 100% [4,7].

Benfaida et al. [7] observed that the most commonly affected anatomical regions were the neck (65.66%), shoulders (63.25%), lumbar region (62.05%), and thoracic region (61.45%). These findings are consistent with other studies, which similarly identify the neck, shoulders, and lower back as the areas most frequently affected. For example, studies conducted in different countries have reported the following prevalences: in Germany, the neck (78.4%), shoulders (66.2%), and lumbar region (58.7%) [3]; in Kuwait, the neck (27.7%), lumbar region (27.1%), and shoulders (22%) [8]; in the United Arab Emirates, the neck (42.6%), lumbar region (36.6%), and shoulders (29.7%) [9]; and in Pakistan, the lumbar region (51.3%), neck (21.3%), and shoulders (17.6%) [10].

As these conditions often begin during training, addressing musculoskeletal health at this stage may be particularly relevant. WMSDs constitute a significant occupational health burden, with potential implications for productivity, workforce sustainability, and long-term career longevity. Preventive strategies implemented during training may therefore contribute to reducing cumulative occupational strain over the professional lifespan.

However, such preventive approaches remain insufficiently integrated into dental education, where structured strategies specifically targeting musculoskeletal health are still limited. Movement-based interventions that promote postural awareness, flexibility, and muscular conditioning have been proposed as potential approaches to mitigate occupational strain. Among these, yoga has gained increasing attention.

Yoga is a multimodal practice that combines physical postures (asanas), breathing exercises, and meditative components [11]. From a physical perspective, yoga-based interventions have been associated with improvements in flexibility, posture, muscular strength, and range of motion, factors that may contribute to reducing mechanical strain in the spine, neck, and shoulders. Clinical trials have reported reductions in musculoskeletal pain, particularly in individuals with chronic neck and low back pain.

In addition to its physical effects, yoga practice has also been investigated in relation to psychological outcomes, including perceived stress and emotional well-being [12,13].

Evidence from dental settings suggests that yoga-based interventions may help address both the musculoskeletal and psychological demands associated with clinical practice [14]. A study conducted among dental professionals reported that 89% of those who practiced yoga were free of pain, compared with 78% of those who engaged in other forms of physical activity [15]. Similarly, research involving dental students has suggested reductions in perceived stress and improvements in well-being following yoga-based interventions.

However, controlled intervention studies examining the role of structured movement-based programmes within postgraduate dental training remain scarce.

The aim of this study was to evaluate whether integrating a structured yoga-based programme within postgraduate dental training was associated with changes in work-related musculoskeletal symptoms among dental trainees. Secondary outcomes included perceived stress and psychological resilience. The study also explored the feasibility of embedding such interventions within training environments as a preventive approach in dental education.

## 2. Materials and Methods

### 2.1. Study Design and Ethical Considerations

This study was designed as a randomized controlled pilot study evaluating the integration of a structured yoga-based intervention within postgraduate dental training. The study was conducted in accordance with relevant reporting guidelines for intervention studies and was approved by the Ethics Committee of Hospital Clínico San Carlos (Madrid, Spain) (No. 24/674-E).

All participants were informed about the objectives of the study and provided written informed consent prior to enrolment, in accordance with the principles of the Declaration of Helsinki.

### 2.2. Participants

A total of 40 postgraduate dental students from the Faculty of Dentistry at the Complutense University of Madrid were included. The sample comprised clinicians from two specialized fields of dentistry: restorative dentistry and orthodontics. Participants were recruited from postgraduate clinical training programmes at the Faculty during the academic year in which the study was conducted.

The sample size was determined based on feasibility considerations and the accessible population, as no prior data were available to support a reliable a priori estimation of effect size.

### 2.3. Inclusion Criteria

- Postgraduate student in dentistry.
- Engagement in at least 20 h of clinical practice per week during the month prior to study initiation.

### 2.4. Exclusion Criteria

- Regular yoga practice for more than one year or within the three months preceding the study.
- Inability to attend scheduled yoga sessions.
- Previous osteomuscular injuries not related to dental practice.
- Medical conditions contraindicating the practice of yoga.
- Previous diagnosis of depression or anxiety, or active treatment for these conditions.

### 2.5. Participant Allocation

- Participants were allocated using computer-generated random numbers and opaque envelopes to ensure allocation concealment and reduce selection bias. One investigator (D.S.) performed the randomization procedure, and a second investigator (C.O.C.) assigned participants to the study groups.
- Experimental group (n = 20): students who participated in the weekly yoga sessions.
- Control group (n = 20): students who did not participate in the yoga sessions but completed all questionnaires at the three evaluation time points.
- The resulting groups differed in the distribution of dental specialties, with the control group consisting predominantly of orthodontic trainees and the intervention group mainly composed of restorative dentistry trainees.

### 2.6. Instruments

The following validated instruments were used:

- Nordic Musculoskeletal Questionnaire (NMQ)

A standardized instrument designed to identify musculoskeletal symptoms in different body regions (neck, shoulders, back, elbows, wrists/hands, hips, knees, and ankles/feet). It assesses the presence of pain or discomfort over the past 12 months and the last 7 days, as well as functional limitations associated with these symptoms [14].

To align symptom assessment with the intervention timeline, the longer recall period was adapted at follow-up assessments to capture symptoms during the preceding 6 weeks (mid-intervention) and 12 weeks (post-intervention), respectively.

For analysis, NMQ responses were recoded into composite scores (0 = no symptoms, 1 = unilateral symptoms, 2 = bilateral symptoms), and total scores were calculated by summing responses across the assessed anatomical regions for each outcome.

- Perceived Stress Scale (PSS-10)

A validated questionnaire comprising 10 items that evaluate the degree to which individuals perceive their lives as stressful during the past month. Each item is rated on a 5-point Likert scale (0 = never to 4 = very often). Total scores range from 0 to 40, with higher values indicating greater perceived stress [12].

- Connor–Davidson Resilience Scale (CD-RISC-10, Spanish version)

A validated 10-item questionnaire assessing the ability to adapt positively to adversity. Responses are rated on a 5-point Likert scale (0 = never to 4 = almost always). Total scores range from 0 to 40, with higher scores indicating greater perceived resilience [16].

### 2.7. Intervention

The intervention consisted of a structured 12-week yoga programme integrated within the training environment. Participants in the experimental group attended one supervised session per week, each lasting approximately 60 min and conducted at the Faculty of Dentistry.

Sessions were delivered by a certified yoga instructor who was also a dentist, allowing adaptation of the exercises to the specific ergonomic demands of dental practice. Each session followed a standardized structure:

- 45 min of physical postures (asanas);
- 10 min of meditation;
- 5 min of breathing exercises (pranayama).

Postures were modified when necessary to avoid excessive strain on the neck, back, wrists, and shoulders, with particular attention to anatomical regions commonly affected by work-related musculoskeletal disorders in dental professionals. The programme was delivered alongside regular clinical training activities.

### 2.8. Procedure

Assessments were conducted at three time points: baseline (week 0), mid-intervention (week 6), and post-intervention (week 12). For musculoskeletal outcomes, the NMQ recall window was adapted at follow-up assessments to reflect the corresponding observation period.

Outcomes were categorized into two main domains:

1. Musculoskeletal outcomes: assessed using the Nordic Musculoskeletal Questionnaire (NMQ);
2. Psychological outcomes: assessed using the Perceived Stress Scale (PSS-10) and the Connor–Davidson Resilience Scale (CD-RISC-10).

The study followed a structured protocol:

- Informed consent and verification of eligibility criteria;
- Random allocation to study groups;
- Baseline assessment (week 0);
- Implementation of the 12-week intervention;
- Mid-intervention assessment (week 6);
- Final assessment (week 12).

Attendance at each yoga session was recorded throughout the intervention period to monitor adherence, and any intervention-related incidents were documented.

### 2.9. Statistical Analysis

Continuous variables are described using means and standard deviations, and categorical variables using frequencies and percentages. Baseline characteristics (age, gender, and handedness) were compared using independent-sample *t*-tests and Pearson’s χ^2^ tests. All participants included in the final sample provided complete outcome data across all assessment time points; therefore, no missing data handling procedures were required. Although assessments were collected at baseline, week 6, and week 12, the primary analyses focused on baseline and post-intervention comparisons to evaluate the overall intervention effect. Changes between groups from baseline to post-intervention were evaluated using repeated-measures ANOVA for normally distributed variables (PSS and CD-RISC) and aligned rank transform ANOVA (ART ANOVA) for NMQ variables, given their non-normal distribution and the high proportion of zero values. Additionally, robust linear models with corrected standard errors were fitted as complementary analyses to evaluate the Group × Time interaction and estimate the magnitude of changes between the intervention and control groups while accounting for heteroscedasticity.

## 3. Results

A total of 40 postgraduate dental students were initially assessed for participation in the study. Three participants were excluded prior to the final study sample: two for health-related reasons and one due to regular yoga practice meeting the exclusion criteria. The final sample included 37 participants, comprising 19 in the control group and 18 in the intervention group. The mean age was 26.7 ± 3.31 years in the intervention group and 25.4 ± 2.17 years in the control group (t = 1.470, *p* = 0.282). Gender distribution did not differ significantly between groups, with 42.1% males and 57.9% females in the intervention group versus 22.2% and 77.8% in the control group (χ^2^ = 0.884, *p* = 0.347). Right-handedness was predominant in both groups (intervention: 89.5%; control: 100%; χ^2^ = 0.473, *p* = 0.491). No statistically significant differences were observed in baseline demographic characteristics. Among participants in the intervention group, mean attendance was 8.1 out of 12 planned sessions (SD = 2.6), with a median attendance of 8.5 sessions (range: 3.5–12). No intervention-related adverse events were reported.

At baseline, the anatomical regions most frequently affected were the neck, upper back, and lower back (Table 1). The Nordic Musculoskeletal Questionnaire items related to symptoms during the previous seven days and work-related impairment were infrequently reported.

**Figure 1:**
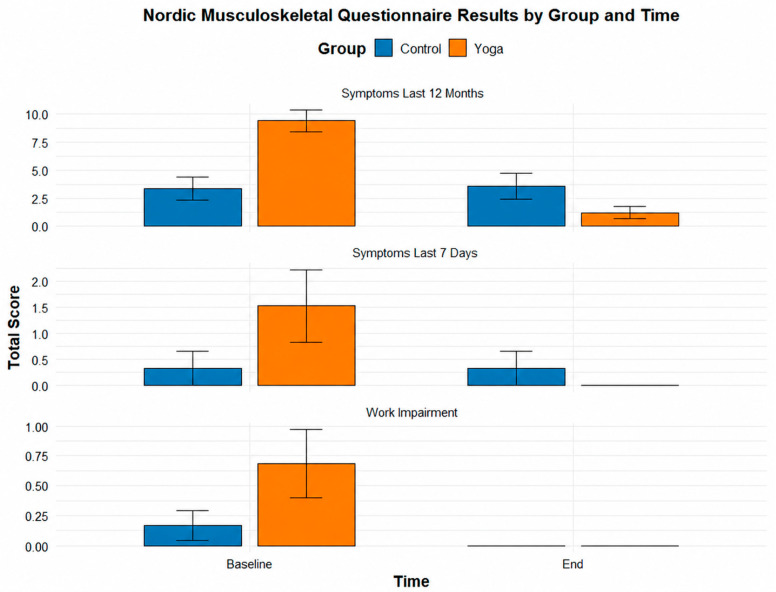
Mean musculoskeletal symptom scores (adapted NMQ recall period and last 7 days) and work-impairment scores by group (yoga vs. control) at baseline and after the 12-week intervention. Error bars represent the standard error of the mean (SEM).

Following the intervention period, musculoskeletal symptom scores decreased in the intervention group while remaining relatively stable in the control group (Figure 1). Differences between groups were identified for symptoms assessed using the adapted NMQ recall period, as well as for symptoms reported during the previous seven days.

Inferential analysis confirmed these patterns (Table 2). ANOVA and aligned rank transform ANOVA (ART ANOVA), applied to NMQ variables due to their non-normal distribution and high proportion of zero values, revealed a significant Group × Time interaction for symptoms assessed using the adapted NMQ recall period (F = 26.879, *p* < 0.001). Complementary robust linear modelling showed an estimated between-group change of −5.59 points (95% CI: −8.02 to −3.15), supporting a greater reduction in the intervention group compared with the control group. For symptoms reported in the previous seven days, the interaction was also significant (F = 5.598, *p* = 0.021). No statistically significant interaction was observed for work impairment (F = 3.889, *p* = 0.053). In an additional descriptive analysis, no new symptom onset was identified among participants asymptomatic at baseline, and symptom worsening was minimal, limited to a small proportion of participants in the adapted NMQ recall measure.

**Figure 2:**
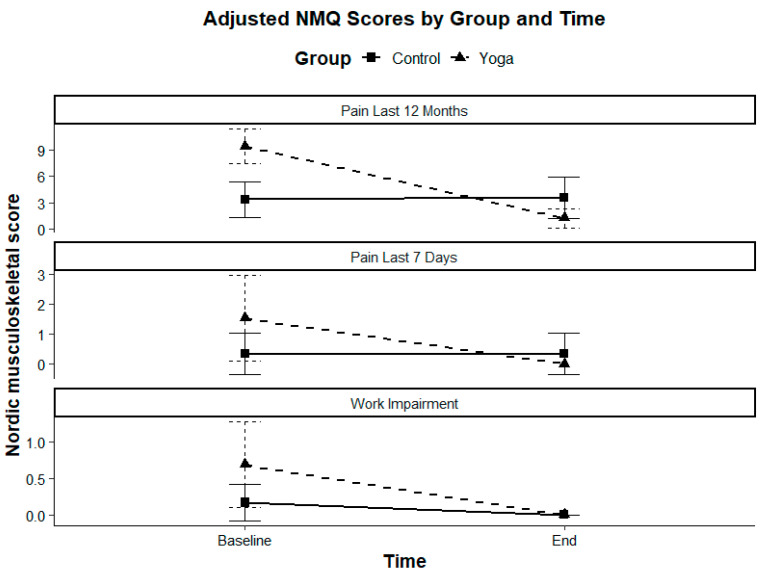
Descriptive trajectories across all three assessment points for musculoskeletal symptom scores (adapted NMQ recall period and last 7 days) and work-impairment scores by group (yoga vs. control). Error bars represent the standard error of the mean (SEM).

For psychological outcomes, no significant Group × Time interaction was observed for the Perceived Stress Scale (PSS) or the Connor–Davidson Resilience Scale (CD-RISC) (*p* = 0.852 and *p* = 0.562, respectively). Although the primary inferential analyses focused on baseline and post-intervention comparisons, Figure 2 presents descriptive trajectory plots across all three assessment points to illustrate temporal patterns over the 12-week period.

## 4. Discussion

This controlled study evaluated the integration of a structured yoga-based programme within postgraduate dental training and its potential to address work-related musculoskeletal symptoms among dental trainees.

Over the past decade, numerous studies have documented the high prevalence of WMSDs among dental professionals, highlighting a persistent occupational health concern that remains insufficiently addressed through preventive strategies [2,3,7,13].

The pattern of musculoskeletal involvement observed in the present study is consistent with previous literature, which identifies the cervical and lumbar regions as the most affected anatomical areas among dental professionals [3,7,8,9,10]. At baseline, the most frequently affected regions were the neck, upper back, and lower back.

Following the intervention, musculoskeletal symptom scores decreased in the intervention group, whereas they remained relatively stable in the control group, consistent with the statistically significant Group × Time interaction observed for the primary musculoskeletal outcome. No comparable effects were identified for psychological measures. However, the intervention group also presented higher baseline musculoskeletal symptom scores, which should be considered when interpreting the observed changes, including the possibility of regression to the mean. The apparent effect was more evident for symptoms assessed using the adapted NMQ recall measure. These observations support a potential role for structured movement-based interventions in addressing musculoskeletal strain associated with dental practice.

Importantly, integrating such interventions within training environments represents a relevant preventive approach, as musculoskeletal strain patterns often develop early during clinical training and may contribute to cumulative occupational burden over the course of a professional career.

Several mechanisms may underlie the observed reduction in musculoskeletal symptoms. In this context, yoga was used as a representative movement-based intervention targeting postural awareness, flexibility, and muscular conditioning. From a biomechanical perspective, yoga involves controlled stretching, strengthening of postural musculature, and increased body awareness, which may reduce mechanical strain and prolonged static loading of the cervical and lumbar regions commonly observed during dental procedures [17].

The present findings are consistent with previous research examining movement-based interventions in dental populations. Koneru et al. [15] reported a lower prevalence of musculoskeletal pain among dentists who practiced yoga compared with those who did not engage in physical activity. Similarly, Monson et al. [13] and Deolia et al. [18] described reductions in musculoskeletal discomfort among dental hygiene students and dental interns following supervised yoga-based programmes.

Regarding psychological outcomes, no significant changes in perceived stress levels were observed following the 12-week intervention. Previous research suggests that psychological responses to yoga interventions may depend on cumulative exposure and intervention frequency. For example, Maddux et al. [19] reported progressively greater reductions in stress and related outcomes in 8- and 16-week programmes, with more pronounced effects observed with longer duration. Similarly, other randomized studies have reported variable effects on perceived stress, influenced by intervention dose and participant adherence [20].

Regarding resilience, a modest increase was observed in the intervention group, although this change did not reach statistical significance. Previous studies suggest that improvements in psychological resilience may require longer intervention periods or higher frequency of practice to become measurable [17,21].

A methodological consideration relates to the adaptation of the NMQ recall period at follow-up assessments to align with the intervention timeline. Although this approach allowed symptom monitoring over the relevant observation periods, modifying the standard recall window may affect direct comparability with studies using the original instrument format.

Differences in clinical practice between dental specialties may also influence musculoskeletal and psychological outcomes. In the present cohort, orthodontic trainees reported slightly lower perceived stress and higher resilience scores at baseline compared with restorative dentistry trainees. Orthodontic practice is often characterized by more predictable treatment schedules, whereas restorative dentistry frequently involves prolonged static postures and technically demanding procedures. These differences in workflow and biomechanical demands may contribute to variations in perceived workload and musculoskeletal strain. However, these differences were not adjusted for in the statistical models and should therefore be interpreted with caution.

Several limitations should be considered when interpreting the present findings. First, the study was conducted within a single academic institution and included a relatively small sample of young postgraduate students, whose greater physical recovery capacity and limited cumulative occupational exposure may have influenced the observed outcomes. Second, the distribution of dental specialties differed between groups, with the control group consisting predominantly of orthodontic trainees and the intervention group mainly including restorative dentistry trainees. Given the distinct ergonomic demands and clinical workloads associated with different specialties, this imbalance may have influenced both baseline symptom burden and outcome trajectories. In addition, the intervention group presented higher baseline musculoskeletal symptom scores, which may have contributed to the observed reduction over time, including the possibility of regression to the mean. Finally, the intervention consisted of one session per week, which may have limited the magnitude of the psychological effects observed. Future studies involving larger samples, longer intervention periods, multicentre designs, and analyses accounting for specialty-related differences are needed to confirm these findings.

## 5. Conclusions

In this pilot sample of postgraduate dental students, participation in a 12-week structured yoga-based programme was associated with reduced work-related musculoskeletal symptoms, whereas no significant changes were observed in perceived stress or resilience. While these findings should be interpreted cautiously, given the small sample size, baseline group differences, and imbalance in dental specialty distribution, they support the potential feasibility of structured movement-based interventions within dental training environments. Larger confirmatory studies are required to further evaluate these findings.

## Figures and Tables

**Table 1:** Baseline distribution of musculoskeletal symptoms by anatomical region according to the Nordic Musculoskeletal Questionnaire (NMQ).

Region	Symptoms Last 12 Months—Yes	Symptoms Last 12 Months—No	Impediment for Job—Yes	Impediment for Job—No	Symptoms Last 7 Days—Yes	Symptoms Last 7 Days—No
Neck	22 (59.5%)	15 (40.5%)	0 (0%)	37 (100%)	4 (10.8%)	33 (89.2%)
Shoulders	12 (32.4%)	25 (67.6%)	0 (0%)	37 (100%)	1 (2.7%)	36 (97.3%)
Upper Back	13 (35.1%)	24 (64.9%)	1 (2.7%)	36 (97.3%)	3 (8.1%)	34 (91.9%)
Elbows	1 (2.7%)	36 (97.3%)	0 (0%)	37 (100%)	0 (0%)	37 (100%)
Lower Back	14 (37.8%)	23 (62.2%)	1 (2.7%)	36 (97.3%)	4 (10.8%)	33 (89.2%)
Wrists	7 (18.9%)	30 (81.1%)	0 (0%)	37 (100%)	0 (0%)	37 (100%)
Hands	9 (24.3%)	28 (75.7%)	1 (2.7%)	36 (97.3%)	0 (0%)	37 (100%)
Hips	5 (13.5%)	32 (86.5%)	0 (0%)	37 (100%)	0 (0%)	37 (100%)
Thighs	0 (0%)	37 (100%)	0 (0%)	37 (100%)	0 (0%)	37 (100%)
Knees	7 (18.9%)	30 (81.1%)	2 (5.4%)	35 (94.6%)	0 (0%)	37 (100%)
Ankles	5 (13.5%)	32 (86.5%)	2 (5.4%)	35 (94.6%)	0 (0%)	37 (100%)
Feet	3 (8.1%)	34 (91.9%)	1 (2.7%)	36 (97.3%)	0 (0%)	37 (100%)

**Table 2:** Mean scores by group and time, and F- and *p*-values for the Group × Time interaction (ANOVA/ART ANOVA).

Test	Yoga Baseline	Yoga End	Control Baseline	Control End	F-Value	*p*-Value
NMQ symptoms (adapted recall)	9.42 (4.14)	1.21 (2.35)	3.33 (4.26)	3.56 (4.96)	26.879	<1 × 10^−4^
Work impairment (NMQ)	0.68 (1.25)	0 (0)	0.17 (0.51)	0 (0)	3.889	0.05254
Symptoms last 7 days (NMQ)	1.53 (3.04)	0 (0)	0.33 (1.41)	0.33 (1.41)	5.598	0.02076
Perceived Stress Scale (PSS)	22.32 (4.19)	22.11 (3.02)	21.89 (5.04)	22.06 (4.81)	0.035	0.8516
Resilience (CD-RISC)	28.74 (4.03)	30.53 (3.6)	32.17 (4.05)	32.89 (4.07)	0.340	0.5619

## Data Availability

The data presented in this study are available within the article. Further information can be requested from the corresponding author.

## Abbreviations

WMSDs	Work-related musculoskeletal disorders
NMQ	Nordic Musculoskeletal Questionnaire
PSS	Perceived Stress Scale
CD-RISC	Connor–Davidson Resilience Scale
